# Endoscopic ultrasound with bronchoscope‐guided transesophageal cryobiopsy: A case series with technical insights, key enablers, and review of literature

**DOI:** 10.1002/rcr2.70082

**Published:** 2024-12-09

**Authors:** Chun Ian Soo, Wai Ling Leong, Diana Bee‐Lan Ong, Lai‐Meng Looi, Leng Cheng Sia, Vijayan Munusamy, Chee Kuan Wong, Nai‐Chien Huan, Hema Yamini Ramarmuty, Khai Lip Ng, Boon Hau Ng, Hazwan Amzar Khairul Annuar, Sze Shyang Kho

**Affiliations:** ^1^ Division of Respiratory Medicine, Department of Medicine University of Malaya Kuala Lumpur Malaysia; ^2^ Department of Biomedical Imaging Universiti Malaya Research Imaging Centre, Universiti Malaya Kuala Lumpur Malaysia; ^3^ Department of Pathology University of Malaya Kuala Lumpur Malaysia; ^4^ Department of Respiratory Medicine Queen Elizabeth Hospital Kota Kinabalu Malaysia; ^5^ Respiratory Unit Hospital Cancelor Tuanku Muhriz UKM Kuala Lumpur Malaysia; ^6^ Division of Respiratory Medicine, Department of Medicine Sarawak General Hospital, Jalan Hospital Kuching Malaysia

**Keywords:** bronchoscopy, cryobiopsy, endobronchial ultrasound, lung cancer

## Abstract

Endobronchial ultrasound‐guided transbronchial needle aspiration (EBUS‐TBNA) is an established technique for lung cancer staging and the diagnosis of mediastinal diseases. Recently, the paradigm of EBUS guided mediastinal sampling with conventional cytology has shifted over to histology specimens through the use of cryobiopsy. This case series explores the novel technique, key enablers, and potential advantages of endoscopic ultrasound with bronchoscope‐guided transesophageal cryobiopsy (EUS‐B‐TEC). The findings of this case series suggest that EUS‐B‐TEC is a safe and valuable addition to the bronchoscopic procedural armamentarium. Further studies are warranted to validate the potential of EUS‐B‐TEC.

## Graphical abstract

A case series on endoscopic ultrasound with bronchoscope‐guided transesophageal cryobiopsy.

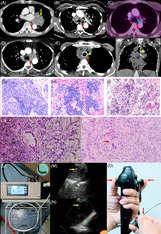

## INTRODUCTION

Endobronchial ultrasound‐guided transbronchial needle aspiration (EBUS‐TBNA) and endoscopic ultrasound with bronchoscope‐guided fine needle aspiration (EUS‐B‐FNA) are commonly used for diagnosis of mediastinal diseases. The use of EBUS scope in both modalities are well‐established for diagnosing and staging of lung cancer.^1^, ^2^ Recently, the incorporation of cryobiopsy to EBUS‐TBNA has gained much attention. The paradigm of mediastinal biopsy sampling with conventional cytology from EBUS‐TBNA has shifted over to histology specimens obtained from Endobronchial ultrasound‐guided transbronchial mediastinal cryobiopsy (EBUS‐TMBC). EBUS‐TBMC allows acquisition of larger and intact tissue samples while preserving cellular architecture, thereby enabling more comprehensive analysis. However, cryobiopsy performed through the transesophageal route (EUS‐B‐guided transesophageal cryobiopsy: EUS‐B‐TEC) is novel. Herein, we describe five interesting cases of EUS‐B‐TEC. For cases involving EUS‐B‐FNA, four needle passes were performed for each lesion with 5 cmH₂O suction applied. All EUS‐B‐TEC cases in this series were conducted using a 22‐gauge needle and the 1.1 mm flexible cryoprobe. For each lesion, we conducted three cryobiopsies with 4 s cryoactivation time. All procedures were under conscious sedation without any major complications. Rapid on‐site evaluation was unavailable for all cases. This case series is followed by a review of the existing literature. Relevant articles on transesophageal cryobiopsy published up to September 30th,2024 were searched using the PubMed database.

## CASE SERIES

**FIGURE 1:**
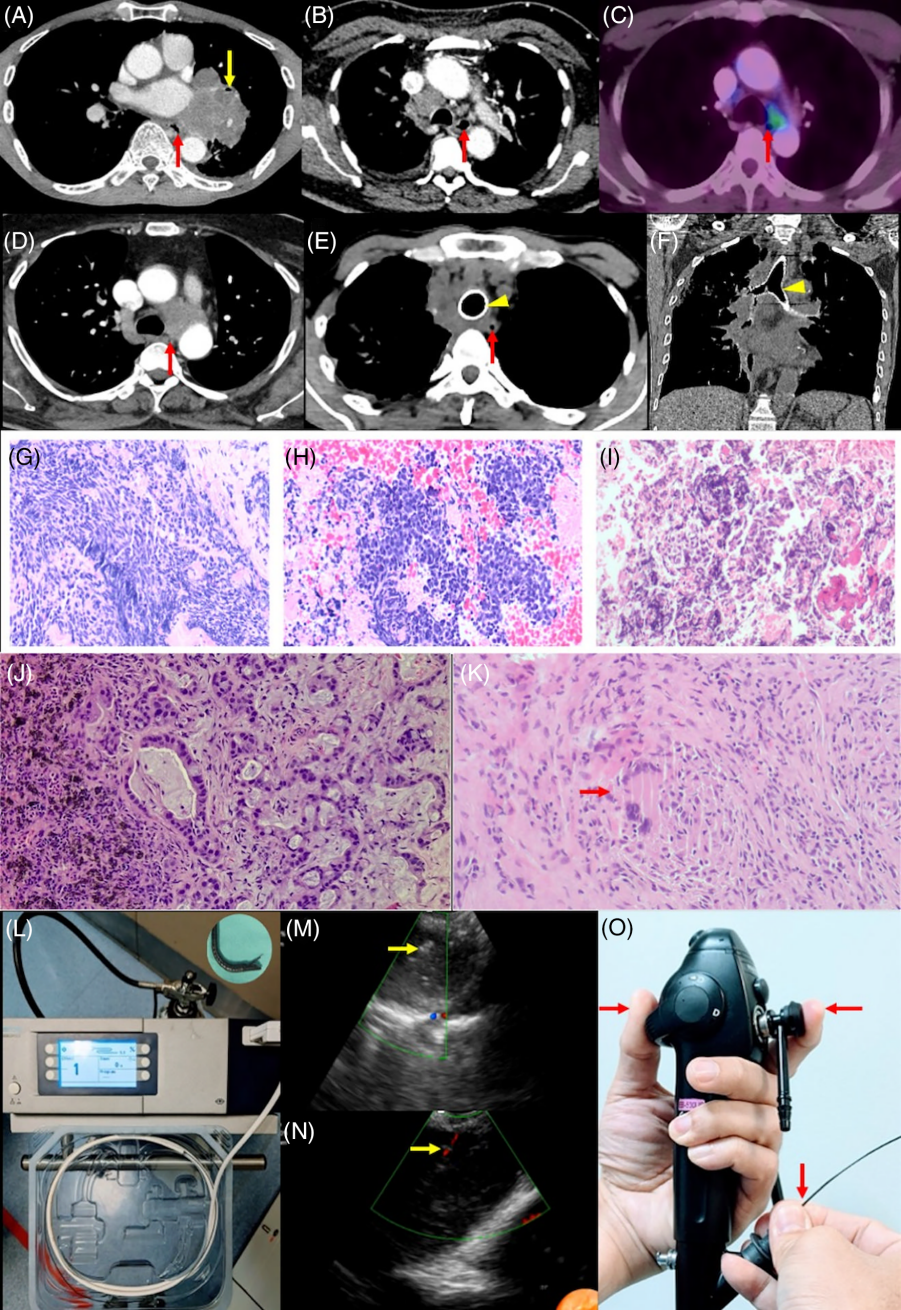
(A) Computed tomography (CT) thorax at the level of the left atrium demonstrating a large left lung mass with narrowing of the left main bronchus (yellow arrow); (B) CT thorax demonstrating enlarged right and left lower paratracheal lymph nodes; (C) PET‐CT in axial view demonstrating hypermetabolic station 4L lymph node (8.2 mm); (D); CT thorax demonstrating a large 4L lymph node, partially compressing the oesophagus with infiltration to the descending thoracic aorta. (E, F) CT thorax in axial and coronal view demonstrating upper paratracheal mass with a silicon Y‐stent (yellow arrowhead); In images A–E, the oesophagus is denoted by the red arrow; (G–I) A comparison of the biopsy specimens obtained from cryobiopsy, tissue cell block and liquid cell block from fine needle aspiration. Microscopic features showing cellular neoplasm of densely packed malignant cells. All three specimens show nests of malignant cells floating in fibrin with malignant cells exhibit high nuclear: Cytoplasmic ratio and hyperchromatic nuclei with nuclear moulding H&E ×100; (J) Abnormal glands with prominent mucin production with residual lymphoid tissue of the lymph node with black carbon pigments H&E ×200 magnification. Results confirmed metastatic adenocarcinoma; (K) Multinucleated giant cells, Langhan type amongst the epithelioid histiocytes and lymphocytes (red arrow); (L) A 1.1 ultrathin flexible cryoprobe (photo inset) attached to a cryosurgery unit; (M) Hyperechoic artefact after needle agitation seen on ultrasonography; (N) Colour doppler signal after needle agitation; (O) Scope handling during EUS‐B‐TEC: Flexion on the EBUS scope level (left thumb), Applying suction through the suction knob (left index finger), firmly hold the cryoprobe (right thumb and index finger) and anchor (right ring and little finger) on the needle adaptor.

Case 1: A 72‐year‐old man with a 50‐pack year smoking history presented with 6 months history of cough and a left‐sided lung mass (7.80 × 6.30 cm) on computed tomography (CT) Thorax. There was no endobronchial lesion and EBUS‐guided biopsy was not possible due to narrowing of the left main bronchus caused by external compression from the mass (Figure 1A). Hence, EUS‐B‐FNA and EUS‐B‐TEC were performed on the left‐sided lung mass. Samples from FNA and TEC were consistent with the diagnosis of small cell lung carcinoma (Figure 1G–I). The patient was commenced on chemotherapy.

Case 2: A 65‐year‐old man presented with a cough for 3 months. He has a 100‐pack‐year smoking history. A CT thorax showed a mass arising from the right hilar region (4.60 × 4.40 × 6.10 cm) associated with multiple mediastinal lymphadenopathies (Figure 1B). In view of multiple oxygen desaturation episodes encountered during EBUS, a decision was made to conduct EUS‐B‐TBNA and EUS‐B‐TEC at stations 4 L and 4R lymph node (LN)s. The cryobiopsy and cytology cellblock results confirmed metastatic lung adenocarcinoma (N3 disease) (Figure 1J). For Next‐generation sequencing (NGS) testing, the cryobiopsy specimen was preferred over cytology cell block by pathologists, as it demonstrated a tumour cell content of 60% compared to 25% in cell block samples. The patient was later commenced on targeted chemotherapy.

Case 3: A 42‐year‐old man who has been in remission for nasopharyngeal carcinoma since 2018 was referred for EBUS‐mediastinal staging for a newly diagnosed left lower lobe lung adenocarcinoma (1.05 × 0.85 cm). A positron emission tomography (PET)‐CT showed hypermetabolic mediastinal lymph nodes at station 4L and 7. A combined EBUS and EUS‐B were planned for comprehensive mediastinal staging. After systematic mediastinal lymph nodes screening, the patient had intractable cough despite maximal sedation (5 mg midazolam and 100 mcg fentanyl). Thus, an EUS‐B‐FNA followed by EUS‐B‐TEC were carried out at station 4L (Figure 1C) and 7 LNs. The histology report revealed lymphoid tissue composed of polymorphous population of lymphocytes admixed with macrophages; some engulfing anthracotic pigments, with no atypical or malignant cells seen. In contrast, the cytology cell block described macrophages containing anthracotic pigments, along with blood, fibrin, lymphocytes, and neutrophils. No granuloma or atypical cells seen. The patient underwent a successful curative lung resection. Mediastinal lymph nodes dissected from station 4L, 5,7,9 and 10L were negative of malignancy.

Case 4: A 35‐year‐old man with type 2 myotonic dystrophy presented with intermittent fever and cough for 6 months. He has multiple matted mediastinal lymphadenopathies. An EUS‐B was preferred over EBUS to avoid compromise in ventilation and EUS‐B‐TEC was favoured over EUS‐B‐FNA in anticipation of a potential case of lymphoma. EUS‐B‐TEC was performed at stations 4L and 7 LNs without EUS‐B‐FNA to minimize overall duration of procedure (Figure 1D). The histology results (Figure 1K) and testing on Xpert MTB/RIF confirmed mycobacterium tuberculosis infection.

Case 5: A 54‐year‐old man was diagnosed and treated for right lung adenocarcinoma (cT4N3M0) 12‐months ago. He also developed central airway obstruction requiring placement of a Silicon Y‐stent (Figure 1E,F). He was referred for a re‐biopsy after a surveillance scan indicated progression of the disease. A successful EUS‐B‐TEC was performed on the right upper paratracheal lesion, yielding adequate specimens to assess patient's eligibility for clinical trials.

## DISCUSSION

The use of EUS‐B by pulmonologists has been well described.^3^, ^4^ In contrast, the application of cryobiopsy guided by EUS‐B is a novel technique that has been less frequently reported. The approach for performing an EUS‐B‐TEC is rather similar to EBUS‐TBMC. The prerequisite for mediastinal cryobiopsy is a biopsy track (BT), which acts as a conduit for the cryoprobe to pass into the target lesion. The first EUS‐B‐TEC report described using the oesophageal air inflation method to enable clear visualization during BT creation with a high‐frequency needle knife followed by the cryoprobe insertion.^5^ Alternatively, the rudimentary method of repeated needle agitations along the same trajectory can be applied by experienced bronchoscopists to create the BT.^6^ The methodology of EUS‐B‐TEC from other reports are shown in Table 1. In our practice, we create the BT by needle agitations alone. Subsequent BT localization and cryoprobe insertion were guided solely by sonographic images and precise recognition of the anatomical landmarks,^9^ as shown in Figure 1M,N. The caveat during cryoprobe insertion for EUS‐B‐TEC is the oesophagus is a mobile, flaccid tubular structure which is distinctly different from the tracheobronchial tree (Video 1). Hence, inserting the cryoprobe can be more challenging and requires fine manoeuvres of the EBUS scope. Occasionally, simultaneous application of suction on the EBUS scope improves sonographic images of the lesion and may facilitate the cryoprobe insertion (Figure 1O). Once the cryoprobe is in situ, the cryosurgery unit (Figure 1L) is cryo‐activated by pressing on a foot pedal. The cryo‐specimen is withdrawn en bloc with the EBUS scope and thawed in room‐temperature saline.

**TABLE 1:** Methodology of published EUS‐B‐TEC articles.

First author	Year	Study site	Design	N	Sampling method	Sedation	Indication for EUS‐B‐TEC	Additional	Complication
Huang^5^	2022	Chongqing, China	Case report	1	TEC after TBNA (21G needle)	Conscious	Lesion accessible via EUS‐B	Oesophageal catheter for Inflation of air into oesophagus High frequency needle knife to created incision	Nil
Ariza‐prota^6^	2023	Oviedo, Spain	Clinical image	1	TEC after TBNA (22‐G needle)	Not reported	Ventilatory failure	Nil	Nil
Salcedo lobera^7^	2024	Malaga, Ovideo, Murcia, Spain	Case series	31	TEC after TBNA (22‐G needle)	Conscious sedation	Lesion accessible via EUS‐B & ventilatory failure	Nil	Nil
Onyancha^8^	2024	Frankfurt, Germany	Retrospective	30	(22‐G needle)	General Anaesthesia	Lesions accessible via EUS‐B	Nil	Nil

Abbreviations: EUS‐B‐TEC, endoscopic ultrasound with bronchoscope‐guided transesophageal cryobiopsy; TBNA, transbronchial needle aspiration.

**VIDEO 1:** Comparison between endobronchial ultrasound‐guided transbronchial mediastinal cryobiopsy (EBUS‐TBMC) versus endoscopic ultrasound with bronchoscope‐guided transesophageal cryobiopsy (EUS‐B‐TEC).

The indications favouring cryobiopsy over needle aspiration in order to improve the diagnostic yield include biopsies for uncommon malignancies and benign conditions such as sarcoidosis and tuberculosis.^10^ In this context, EUS‐B‐TEC is a reasonable alternative when EBUS is not feasible due to poor respiratory reserves or limited patient tolerance. In addition, EUS‐B‐TEC is the preferred approach for targeting lesions that are inaccessible to EBUS such as the inferior mediastinal lymph nodes, para‐oesophageal and para‐vertebral lesions.

Another key indication for considering EUS‐B‐TEC is the presence of central airway narrowing. This is highlighted in first case where EBUS was not feasible due to the critically narrowed main bronchus. In the second and third case, EUS‐B‐TEC served as an alternative when there were safety concerns regarding ventilation and patient tolerance towards EBUS. Poor tolerance to EBUS is common as the tracheobronchial tree contains highly sensitive sensory innervation. Poor tolerance may also result from difficulty in providing optimal sedation, especially in patients with poor ventilatory reserves. In patients with increased intracranial pressure, poor tolerance during the procedure causes sympathetic stimulation, raised pulmonary intravascular pressure and pulmonary edema.^11^ In addition, respiratory acidosis and hypercapnia during EBUS‐TBNA causes vasodilation and could further elevate intracranial pressure. Hence, available evidence suggesting EUS‐B‐FNA provides a better tolerance,^7^ can be extrapolated for EUS‐B‐TEC. In case number four, EUS‐B‐TEC obviates the risk of general anaesthesia and the necessity of complex hemodynamic and cerebral oximetry monitoring in a patient with neuromuscular disorder. It was well tolerated by the patient.

The oesophagus lies between the trachea and the vertebra bodies before entering the abdomen through the oesophagus hiatus. Lymph nodes, lung or mediastinal lesions at the left paratracheal, subcarinal, and large right paratracheal lesions are accessible for EUS‐B‐TEC. Extended access to lymph nodes at stations^10^, ^11^ and the left adrenal gland is possible for complete mediastinal staging for lung cancer. Huang et al. described a successful biopsy of a mediastinal lesion using EUS‐B‐TEC when EBUS was not accessible.^5^ Similarly, in case number five, the EUS‐B‐TEC approach was used to obtain histology specimens from an anatomical area that could be safely biopsied without interfering with the airway stent.

In terms of diagnostic yield, a limited number of case series have reported higher diagnostic yield from cryobiopsy over needle aspiration, especially in cases of lymphoma and benign disorders.^6^, ^7^ In our case series, histology specimens from EUS‐B‐TEC are noticeably far more cellular and demonstrated good tissue architecture (Figure 1G–I). For non‐small cell carcinoma, although cytology specimen from EUS‐B‐FNA could possibly provide adequate samples for NGS testing,^12^ higher tumour cell burden from histology specimens is generally favoured over cytology cell blocks for NGS testing. For mediastinal staging, histology specimens from EUS‐B‐TEC could provide greater certainty in excluding metastasis compared to the cytology specimens as shown in case number three.

The safety of cryobiopsy via the oesophagus raises valid concerns, particularly regarding the risk of oesophageal rupture. However, existing literature on oesophageal perforation primarily highlights incidents were linked to various therapeutic endoscopic procedures such as dilatation manoeuvres, removal of foreign body or application of oesophageal endoprostheses.^13^, ^14^ Incidence of oesophageal perforation secondary to purely diagnostic endoscopy procedures is less than 1%.^13^, ^14^ While the risk of laceration or perforation with cryobiopsy should not be dismissed entirely, it is generally considered to be relatively low when proper techniques are employed. Although there is a paucity of data on the safety and complications of EUS‐B‐TEC, other potential complications following EUS‐B‐TEC including bleeding post biopsy, pneumomediastinum and pneumothorax may be similar to those observed with EBUS‐TBMC. In a meta‐analysis, self‐limiting mild bleeding following TBMC was reported in 36.5% (202 patients) of cases, while severe bleeding occurred in up to 0.7% (4 patients) of cases, requiring bronchoscopic intervention.^10^ In our case series, we observed only mild degree of bleeding post‐EUS‐B‐TEC in the first case. To mitigate the risk of bleeding, some crucial precautionary measures should include the use of colour flow Doppler to avoid targeting vascular areas, minimizing cryoactivation time, and applying minimal force during the insertion and retraction of the cryoprobe. In addition, the risk of mediastinitis shouldn't be overlooked, given that the oesophagus is a non‐sterile cavity. However, the incidence of this complication remains largely unknown. We postulate that the loss of integrity of the transesophageal BT after the procedure limits communication between the oesophagus and the mediastinum and could play a significant role in reducing such complications. Avoiding cryobiopsies in areas exhibiting necrotic or cystic appearances could also help to reduce the risk of complications.^15^

In conclusion, EUS‐B‐TEC is a valuable tool in evaluating mediastinal diseases and staging lung cancer. EUS‐B‐TEC is well tolerated, safe and can be utilized as an ancillary tool for EUS‐B‐FNA and EBUS‐TBMC. Further studies are needed to establish its role before permanently adopting this new procedure into our clinical practice.

## AUTHOR CONTRIBUTIONS

*Conceptualization and design*: Chun Ian Soo, Nai‐Chien Huan, Boon Hau Ng. *Collection and assembly of data*: Chun Ian Soo, Wai Ling Leong, Diana Bee‐Lan Ong, Lai‐Meng Looi, Hema Yamini Ramarmuthy, Khai Lip Ng, Leng Cheng Sia, Vijayan Munusamy, Hazwan Amzar Khairul Annuar. *Writing‐original draft*: Chun Ian Soo, Wai Ling Leong, Boon Hau Ng. *Review, editing and supervision*: Chun Ian Soo, Chee Kuan Wong, Nai‐Chien Huan, Sze Shyang Kho. All authors have approved the submitted version of the manuscript and agreed to be accountable for any part of the work.

## FUNDING INFORMATION

No funding was received for this report.

## CONFLICT OF INTEREST STATEMENT

Dr. Sze Shyang Kho has received travel grant from ERBE Elektromedizin GmbH. Dr. Chun Ian Soo has received speaker honoraria from Cook Medical. All other authors have no conflict of interest.

## ETHICS STATEMENT

The authors declare that appropriate written informed consent was obtained for the publication of this manuscript and accompanying images. The study was approved by the Institutional Ethics Committee of University of Malaya (2024418‐13645; approved 9th May 2024) and all participants gave written informed consent.

## Data Availability

Data sharing not applicable to this article as no datasets were generated or analysed during the current study.

## References

[1] Detterbeck FC , Jantz MA , Wallace M , Vansteenkiste J , Silvestri GA . Invasive mediastinal staging of lung cancer: ACCP evidence‐based clinical practice guidelines. Chest. 2007;132:202S–220S. 17873169 10.1378/chest.07-1362

[2] Vilmann P , Clementsen PF , Colella S , Siemsen M , De Leyn P , Dumonceau JM , et al. Combined endobronchial and esophageal endosonography for the diagnosis and staging of lung cancer: European Society of Gastrointestinal Endoscopy (ESGE) Guideline, in cooperation with the European Respiratory Society (ERS) and the European Society of Thoracic Surgeons (ESTS). Endoscopy. 2015;47:545–559. 26030890 10.1055/s-0034-1392040

[3] Clementsen PF , Bodtger U , Konge L , Christiansen IS , Nessar R , Salih GN , et al. Diagnosis and staging of lung cancer with the use of one single echoendoscope in both the trachea and the esophagus: a practical guide. Endosc Ultrasound. 2021;10:325–334. 33666182 10.4103/EUS-D-20-00139 PMC8544013

[4] Hwangbo B , Lee HS , Lee GK , Lim KY , Lee SH , Kim HY , et al. Transoesophageal needle aspiration using a convex probe ultrasonic bronchoscope. Respirology. 2009;14:843–849. 19659830 10.1111/j.1440-1843.2009.01590.x

[5] Huang ZS , Zhou D , Zhang J , Fu WL , Wang J , Wu XL , et al. Mediastinal nodular lymphocyte predominant Hodgkin lymphoma achieved by endoscopic transesophageal cryobiopsy. Respiration. 2022;101:190–194. 34515245 10.1159/000518598

[6] Ariza‐Prota MA , de Santis M , López‐González F . Successful diagnostic mediastinal cryobiopsy by transesophageal endoscopy without using the needle knife. Arch Bronconeumo. 2023;59:601–602. 10.1016/j.arbres.2023.06.014 37495443

[7] Lobera ES , Prota MA , Pallarés JP , González FL , Codeso FP . Transesophageal endoscopic ultrasound‐guided mediastinal Cryobiopsy in the diagnosis of mediastinal lesions: our experience in 31 cases. Arch Bronconeumo. 2024;60(9):587–589. 10.1016/j.arbres.2024.05.005 38811320

[8] Onyancha S , Nitsch E , Tekeli‐Camci N , Dedeoglu B , Kiil K , Rohde G . Feasibility and safety of transesophageal mediastinal Cryobiopsy in the diagnosis of mediastinal pathologies. Respiration. 2024;103:1–6. 39173596 10.1159/000541084

[9] Soo CI , Huan NC , Kho SS . Technical tips, diagnostic yield and safety of endobronchial ultrasound‐guided transbronchial mediastinal cryobiopsy. Med J Malaysia. 2024;79:490–493. 39086350

[10] Mathew R , Roy WE , Thomas ES , Meena N , Danilevskaya O . Meta‐analysis and systematic review of mediastinal cryobiopsy versus endobronchial ultrasound‐transbronchial needle aspiration (EBUS‐TBNA) in the diagnosis of intrathoracic adenopathy. J Thorac Dis. 2024;16(7):4217–4228. 39144333 10.21037/jtd-24-348 PMC11320235

[11] Harrison W , Liebow AA . The effects of increased intracranial pressure on the pulmonary circulation in relation to pulmonary edema. Circulation. 1952;5:824–832. 14936178 10.1161/01.cir.5.6.824

[12] Himeji D , Shiiba R , Tanaka GI , Takano A , Kamiike R , Kushima N , et al. Usefulness of endoscopic ultrasound with bronchoscope‐guided fine‐needle aspiration for next‐generation sequencing in patients with non‐small cell lung cancer: a comparison with other bronchoscopic techniques. Respir Investig. 2024;62(5):879–883. 10.1016/j.resinv.2024.07.012 39096541

[13] Chirica M , Champault A , Dray X , Sulpice L , Munoz‐Bongrand N , Sarfati E , et al. Esophageal perforations. J Visc Surg. 2010;147(3):e117–e128. 20833121 10.1016/j.jviscsurg.2010.08.003

[14] Lampridis S , Mitsos S , Hayward M , Lawrence D , Panagiotopoulos N . The insidious presentation and challenging management of esophageal perforation following diagnostic and therapeutic interventions. J Thorac Dis. 2020;12(5):2724–2734. 32642181 10.21037/jtd-19-4096 PMC7330325

[15] Romero AO , Kho SS . Transbronchial mediastinal cryobiopsy—literature review and practice recommendations. AME Med J. 2024;9:9.

